# Supplementary material for: Evaluating the feasibility of medium-chain oleochemical synthesis using microbial chain elongation
Source: J Ind Microbiol Biotechnol. 2024 Aug 1;51:kuae027. doi: 10.1093/jimb/kuae027 (PMC11388927; doi:10.1093/jimb/kuae027)
Supplement: kuae027_Supplemental_Files [file kuae027_supplemental_files.zip › Supplemental Material S1 Supplementary Text.pdf]

# Supplemental Material

## Evaluating the feasibility of medium chain oleochemical synthesis using microbial chain elongation

Ethan Agena[1,#], Ian M. Gois[1,#], Connor M. Bowers[1,#], Radhakrishnan Mahadevan[1], Matthew J. Scarborough[2], Christopher E. Lawson[1*]

[1]Department of Chemical Engineering & Applied Chemistry, University of Toronto, Toronto, ON, Canada

[2]Department of Civil and Environmental Engineering, University of Vermont, Burlington, VT, USA

[#]These authors contributed equally to this work

*Correspondence should be addressed to: Christopher E. Lawson. E-mail: chris.lawson@utoronto.ca

## Table of Contents

***Production of $C_4$-$C_{12}$ Carboxylates*** ... 2

***Resource Allocation Model Formulation*** ... 5

***ECM Free Energy Changes*** ... 7

***Anaerobic Oxidation of Heptanoic Acid to Adipate*** ... 8

## Production of $C_4$-$C_{12}$ Carboxylates

Table 1: Predicted stoichiometry, ATP Yield, and free energy of reaction for the synthesis of $C_4$-$C_{12}$ carboxylates from lactate

| Substrate | Product | Chain Length | Overall Equation | ATP Yield (mol / mol Lactate) | dG0' (kJ/mol) | dG0' per ATP (kJ/mol / mol ATP) |
|---|---|---|---|---|---|---|
| Lactate | Carboxylate | C4 | Lactate + 0.5 $H^+$ + 0.25 ADP → 0.5 Butyrate + $CO_2$ + $H_2$ + 0.25 ATP | 0.25 | -14.989 | -59.957 |
| Lactate | Carboxylate | C6 | Lactate + 0.667 $H^+$ + 0.333 ADP → $CO_2$ + 0.667 $H_2$ + 0.333 $H_2O$ + 0.333 Hexanoate + 0.333 ATP | 0.333 | -26.746 | -80.239 |
| Lactate | Carboxylate | C8 | Lactate + 0.75 $H^+$ + 0.375 ADP → $CO_2$ + 0.5 $H_2$ + 0.5 $H_2O$ + 0.25 Octanoate + 0.375 ATP | 0.375 | -35.101 | -93.603 |
| Lactate | Carboxylate | C10 | Lactate + 0.8 $H^+$ + 0.4 ADP → $CO_2$ + 0.2 Decanoate + 0.4 $H_2$ + 0.6 $H_2O$ + 0.4 ATP | 0.400 | -40.114 | -100.285 |
| Lactate | Carboxylate | C12 | Lactate + 0.833 $H^+$ + 0.417 ADP → $CO_2$ + 0.167 Dodecanoate + 0.333 $H_2$ + 0.667 $H_2O$ + 0.417 ATP | 0.417 | -43.456 | -104.294 |

Table 2: Predicted stoichiometry, ATP Yield, and free energy of reaction for the synthesis of $C_4$-$C_{12}$ carboxylates from ethanol

| Substrate | Product | Chain Length | Overall Equation | ATP Yield (mol / mol Lactate) | dG0' (kJ/mol) | dG0' per ATP (kJ/mol / mol ATP) |
|---|---|---|---|---|---|---|
| Ethanol | Carboxylate | C4 | Ethanol + 0.25 ADP → 0.5 Butyrate + 0.5 $H^+$ + $H_2$ + 0.25 ATP | 0.25 | 0.481 | 1.923 |
| Ethanol | Carboxylate | C6 | Ethanol + 0.333 ADP → 0.333 $H^+$ + 0.667 $H_2$ + 0.333 $H_2O$ + 0.333 Hexanoate + 0.333 ATP | 0.333 | -11.277 | -33.83 |
| Ethanol | Carboxylate | C8 | Ethanol + 0.375 ADP → 0.25 $H^+$ + 0.5 $H_2$ + 0.5 $H_2O$ + 0.25 Octanoate + 0.375 ATP | 0.375 | -19.631 | -52.35 |
| Ethanol | Carboxylate | C10 | Ethanol + 0.4 ADP → 0.2 Decanoate + 0.2 $H^+$ + 0.4 $H_2$ + 0.6 $H_2O$ + 0.4 ATP | 0.4 | -24.644 | -61.61 |
| Ethanol | Carboxylate | C12 | Ethanol + 0.417 ADP → 0.167 Dodecanoate + 0.167 $H^+$ + 0.333 $H_2$ + 0.667 $H_2O$ + 0.417 ATP | 0.417 | -27.986 | -67.166 |

Table 3: Predicted stoichiometry, ATP Yield, and free energy of reaction for the synthesis of $C_4$-$C_{12}$ carboxylates from glucose

| Substrate | Product | Chain Length | Overall Equation | ATP Yield (mol / mol Lactate) | dG0' (kJ/mol) | dG0' per ATP (kJ/mol / mol ATP) |
|---|---|---|---|---|---|---|
| Glucose | Carboxylate | C4 | Glucose + 3.6 ADP → 1.2 Butyrate + 1.2 $CO_2$ + 1.2 $H^+$ + 1.2 $H_2O$ + 3.6 ATP | 3.6 | -304.531 | -84.592 |
| Glucose | Carboxylate | C6 | Glucose + 3.75 ADP → 1.5 $CO_2$ + 0.75 $H^+$ + 1.5 $H_2O$ + 0.75 Hexanoate + 3.75 ATP | 3.75 | -296.975 | -79.193 |
| Glucose | Carboxylate | C8 | Glucose + 3.818 ADP → 1.636 $CO_2$ + 0.545 $H^+$ + 1.636 $H_2O$ + 0.545 Octanoate + 3.818 ATP | 3.818 | -298.943 | -78.295 |
| Glucose | Carboxylate | C10 | Glucose + 3.857 ADP → 1.714 $CO_2$ + 0.429 Decanoate + 0.429 $H^+$ + 1.714 $H_2O$ + 3.857 ATP | 3.857 | -300.068 | -77.795 |
| Glucose | Carboxylate | C12 | Glucose + 3.882 ADP → 1.765 $CO_2$ + 0.353 Dodecanoate + 0.353 $H^+$ + 1.765 $H_2O$ + 3.882 ATP | 3.882 | -300.795 | -77.478 |

Table 4: Predicted stoichiometry, ATP Yield, and free energy of reaction for the synthesis of $C_4$-$C_{12}$ carboxylates from xylose

| Substrate | Product | Chain Length | Overall Equation | ATP Yield (mol / mol Lactate) | dG0' (kJ/mol) | dG0' per ATP (kJ/mol / mol ATP) |
|---|---|---|---|---|---|---|
| Xylose | Carboxylate | C4 | D-Xylose + 3.0 ADP → Butyrate + $CO_2$ + $H^+$ + $H_2O$ + 3.0 ATP | 3 | -266.531 | -88.844 |
| Xylose | Carboxylate | C6 | D-Xylose + 3.125 ADP → 1.25 $CO_2$ + 0.625 $H^+$ + 1.25 $H_2O$ + 0.625 Hexanoate + 3.125 ATP | 3.125 | -260.235 | -83.275 |
| Xylose | Carboxylate | C8 | D-Xylose + 3.182 ADP → 1.364 $CO_2$ + 0.455 $H^+$ + 1.364 $H_2O$ + 0.455 Octanoate + 3.182 ATP | 3.182 | -261.875 | -82.303 |
| Xylose | Carboxylate | C10 | D-Xylose + 3.214 ADP → 1.429 $CO_2$ + 0.357 Decanoate + 0.357 $H^+$ + 1.429 $H_2O$ + 3.214 ATP | 3.214 | -262.812 | -81.764 |
| Xylose | Carboxylate | C12 | D-Xylose + 3.235 ADP → 1.471 $CO_2$ + 0.294 Dodecanoate + 0.294 $H^+$ + 1.471 $H_2O$ + 3.235 ATP | 3.235 | -263.418 | -81.42 |

Table 5: Predicted stoichiometry, ATP Yield, and free energy of reaction for the synthesis of $C_4$-$C_{12}$ carboxylates from glycerol

| Substrate | Product | Chain Length | Overall Equation | ATP Yield (mol / mol Lactate) | dG0' (kJ/mol) | dG0' per ATP (kJ/mol / mol ATP) |
|---|---|---|---|---|---|---|
| Glycerol | Carboxylate | C4 | Glycerol + 1.25 ADP → 0.5 Butyrate + $CO_2$ + 0.5 $H^+$ + 2.0 $H_2$ + 1.25 ATP | 1.25 | -53.307 | -42.646 |
| Glycerol | Carboxylate | C6 | Glycerol + 1.333 ADP → $CO_2$ + 0.333 $H^+$ + 1.667 $H_2$ + 0.333 $H_2O$ + 0.333 Hexanoate + 1.333 ATP | 1.333 | -65.064 | -48.798 |
| Glycerol | Carboxylate | C8 | Glycerol + 1.375 ADP → $CO_2$ + 0.25 $H^+$ + 1.5 $H_2$ + 0.5 $H_2O$ + 0.25 Octanoate + 1.375 ATP | 1.375 | -73.419 | -53.396 |
| Glycerol | Carboxylate | C10 | Glycerol + 1.4 ADP → $CO_2$ + 0.2 Decanoate + 0.2 $H^+$ + 1.4 $H_2$ + 0.6 $H_2O$ + 1.4 ATP | 1.4 | -78.432 | -56.023 |
| Glycerol | Carboxylate | C12 | Glycerol + 1.417 ADP → $CO_2$ + 0.167 Dodecanoate + 0.167 $H^+$ + 1.333 $H_2$ + 0.667 $H_2O$ + 1.417 ATP | 1.417 | -81.774 | -57.723 |

**Resource Allocation Model Formulation**

A simple steady state balance on ATP per unit biomass ($[ATP]$) yields a model which reflects (1) the expectation that a higher growth rate requires a larger pool of biosynthetic enzymes and ribosomes, (2) a higher growth rate requires a faster specific ATP flux and (3) the catalytic capacity of a cell is finite.

$$\frac{d[ATP]}{dt} = J_{cat} + J_{ana} - \lambda[ATP] - b = 0$$

Where $J_{cat}$ is the specific ATP production flux (ATP equivalents per cell mass per time), $J_{ana}$ is the specific ATP consumption flux for growth, $\lambda$ is the specific growth rate and $b$ is the specific flux of ATP for maintenance/decay. We assume that $J_{cat}$ and $J_{ana}$ are linearly related to the fraction of cell mass allocated to catabolism and anabolism, $\phi_{cat}$ and $\phi_{ana}$ respectively. $\Lambda/N_{ATP}$ is the enzyme investment per unit flux through the cell's catabolic pathway, in units of enzyme mass per substrate equivalents per time, divided by the pathways ATP yield (ATP equivalents per substrate equivalents). $\gamma$ relates the fraction of biomass engaged in anabolic catalysis to the specific ATP consumption flux and has units of ATP equivalents per anabolic mass per time.

$$J_{cat} = \frac{\phi_{cat}}{\Lambda/N_{ATP}}$$

$$J_{ana} = \gamma\phi_{ana}$$

$\phi_0$ is the remaining non-catalytic cell mass fraction, which is assumed to remain constant at 20% of the cell's mass.

$$\phi_{cat} + \phi_{ana} + \phi_0 = 1$$

$Y_{M/ATP}$ is the cell mass yield on ATP (cell mass produced per ATP equivalents consumed).

$$\lambda = \frac{1}{M}\frac{dM}{dt} = Y_{M/ATP} \cdot J_{ana}$$

This system yields the following relationship between growth rate and enzyme cost per ATP flux:

$$\frac{\Lambda}{N_{ATP}} = \left(\frac{1-\phi_0}{\lambda} + \frac{1}{\gamma Y_{M/ATP}}\right) \Bigg/ \left(\frac{1}{Y_{M/ATP}} + [ATP] + \frac{b}{\lambda}\right)$$

Maintenance energy and dilution of the ATP pool due to growth are assumed to be negligeable, leaving:

$$\frac{\Lambda}{N_{ATP}} = \frac{Y_{M/ATP}(1-\phi_0)}{\lambda} + \frac{1}{\gamma}$$

And:

$$\phi_{cat} = 1 + \frac{\lambda}{\gamma Y_{M/ATP}} - \phi_0$$

To serve as a qualitative comparison between product cases, the enzyme cost per ATP flux and growth rate of a butyrate producer is set to 1, and the fraction of catabolic biomass is set to 0.1, allowing for the parametrization of $\gamma$ and $Y_{M/ATP}$ in arbitrary units (10 and 0.14 respectively).

## ECM Free Energy Changes

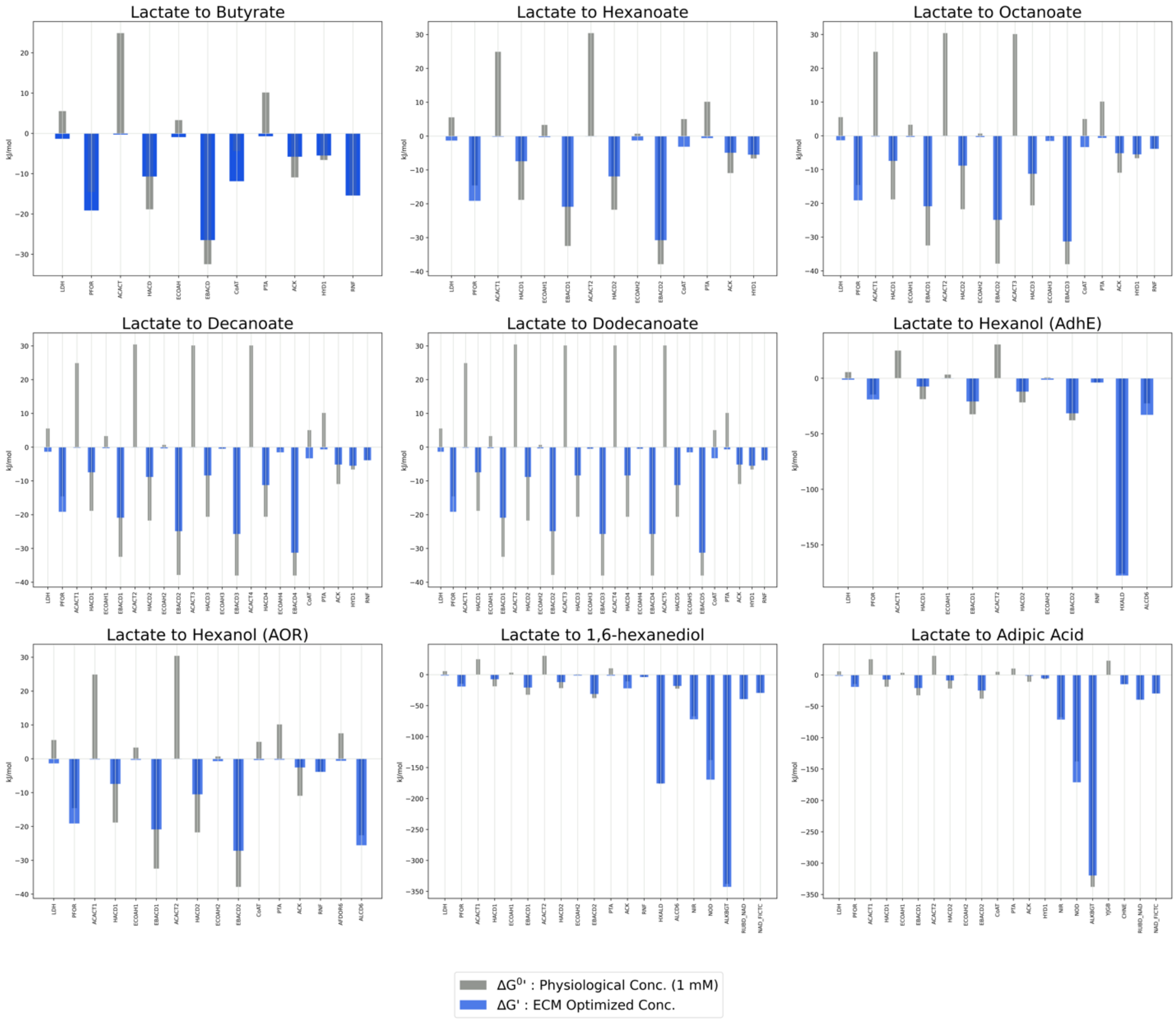


Supplementary Figure 1. Reaction Gibbs free energy changes in chain elongation pathways predicted by Enzyme Cost Minimization (Flamholz et al. 2013). Physiological ΔG°' (grey) are evaluated at uniform metabolite concentrations of 1 mM. Optimized ΔG' follow from the set of metabolite concentrations which minimizes enzyme demand per unit pathway flux. All are evaluated at pH 6 and 25 °C.

**Anaerobic Oxidation of Heptanoic Acid to Adipate**

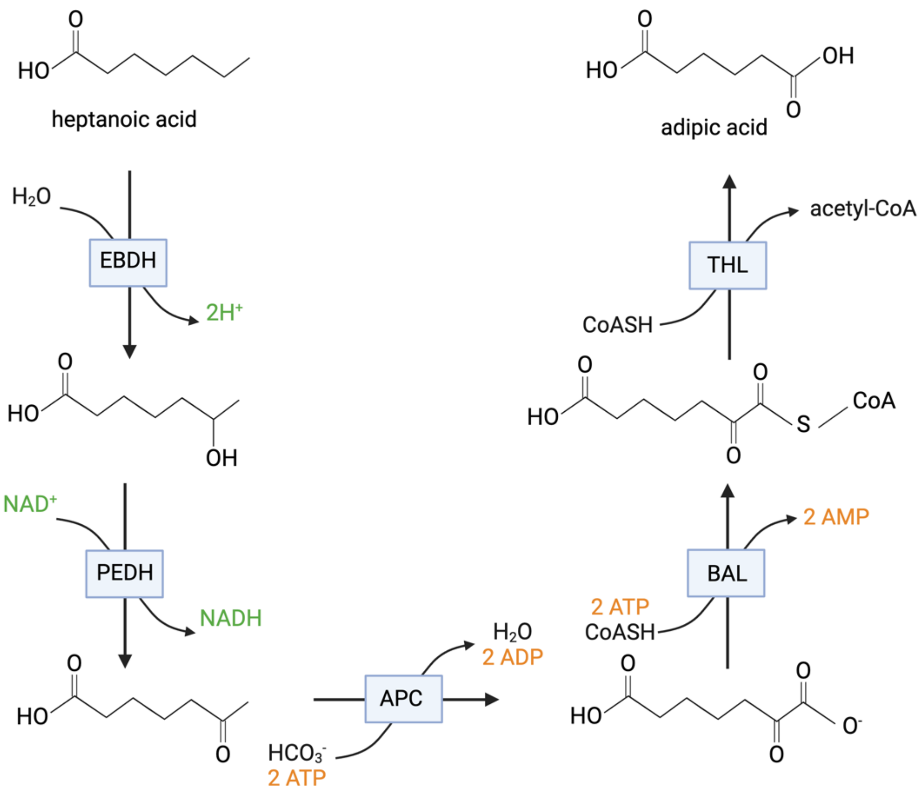


Supplementary Figure 2. Hypothetical anaerobic oxidation of heptanoic acid to adipic acid. Adapted from Heider et al., 2016. EBDH: ethylbenzene dehydrogenase; PEDH: phenylethanol dehydrogenase; APC: acetophenone carboxylase; BAL: benzoylacetate-CoA ligase; THL: thiolase. Created with BioRender.com
